# Nature-based solutions for atoll habitability

**DOI:** 10.1098/rstb.2021.0124

**Published:** 2022-07-04

**Authors:** Jon Barnett, Sergio Jarillo, Stephen E. Swearer, Catherine E. Lovelock, Andrew Pomeroy, Teresa Konlechner, Elissa Waters, Rebecca L. Morris, Ryan Lowe

**Affiliations:** ^1^ Geography, Earth and Atmospheric Sciences, The University of Melbourne, Melbourne, Victoria 3010, Australia; ^2^ National Centre for Coasts and Climate, School of BioSciences, The University of Melbourne, Melbourne, Victoria 3010, Australia; ^3^ School of BioSciences, The University of Melbourne, Melbourne, Victoria 3010, Australia; ^4^ School of Biological Sciences, The University of Queensland, St Lucia, Queensland 4072, Australia; ^5^ Wildlife Consultants Ltd, 7A Vulcan Place, Middleton, Christchurch 8024, New Zealand; ^6^ Oceans Graduate School, and School of Earth Sciences, The University of Western Australia, Perth, Western Australia 6009, Australia

**Keywords:** adaptation, engineering, islands, social dimensions, technology, tropics

## Abstract

Atoll societies have adapted their environments and social systems for thousands of years, but the rapid pace of climate change may bring conditions that exceed their adaptive capacities. There is growing interest in the use of ‘nature-based solutions' to facilitate the continuation of dignified and meaningful lives on atolls through a changing climate. However, there remains insufficient evidence to conclude that these can make a significant contribution to adaptation on atolls, let alone to develop standards and guidelines for their implementation. A sustained programme of research to clarify the potential of nature-based solutions to support the habitability of atolls is therefore vital. In this paper, we provide a prospectus to guide this research programme: we explain the challenge climate change poses to atoll societies, discuss past and potential future applications of nature-based solutions and outline an agenda for transdisciplinary research to advance knowledge of the efficacy and feasibility of nature-based solutions to sustain the habitability of atolls.

This article is part of the theme issue ‘Nurturing resilient marine ecosystems’.

## 1. Introduction

Atolls are dynamic environments that are highly exposed to impacts from climate change, and while considerable effort has been invested in asserting that they are highly endangered by climate change, very little effort has been invested in comprehensively explaining exactly how climate change will drive changes in atoll environments, and in exploring solutions to ensure their habitability [1]. Habitability refers to the conditions under which people are able to lead dignified and meaningful lives characterized by satisfactory human development, livelihoods, and well-being [2,3]. It is a novel issue brought into being by the rapid pace of climate change, which threatens to progressively reduce peoples’ access to the proximate environmental goods and services on which their lives depend (in most atolls this dependence is very high). In this context nature-based solutions can in theory make important contributions to climate change adaptation on atolls by working with and enhancing the ecosystems that sustain people's access to environmental goods and services [4].

Globally, there is growing enthusiasm for the use of nature-based solutions to support climate change mitigation and adaptation, but almost none of the existing research helps inform their application to sustain the habitability of atolls. The focus of most of the research and practice has been on conserving and increasing terrestrial vegetation in cities and degraded lands to achieve biodiversity, carbon mitigation and social co-benefits [5,6]. There is a lesser but growing interest in green infrastructure to manage coastal erosion and inundation in ways that also promote biodiversity conservation, carbon mitigation and livelihood co-benefits [7–10]. Yet for the most part research on nature-based solutions in the coastal zone has focused on studies seeking to test their technical feasibility in temperate developed countries. However, their real-world efficacy in these and other parts of the world will be a function of many other factors including their cost, governance and social acceptability [11–13].

Thus, to date, there has been very little research and even less practical application of nature-based solutions in the unique social-ecological environments of atolls. This deficit between the imperative for nature based-solutions to support climate change adaptation on atolls and peer-reviewed evidence to inform their application is growing, and unless remedied the potential for nature-based solutions will remain unrealized or, worse, applications will be maladaptive and shunned by investors and local people alike. This paper seeks to begin to fill this gap. It offers a prospectus to guide research to understand the potential of nature-based solutions to support the habitability of rural atoll islands. Given the small scale of these islands and the costs associated with their remoteness, nature-based solutions will most likely be similarly small in scale and applied to protect key local sites such as villages, or critical facilities and sites such as cemeteries, gardens, harbours, health centres, landing strips and schools. In the next section, we explain the challenge climate change poses to the habitability of atoll societies, and discuss past applications of what might be called ‘nature-based solutions'. We then outline an agenda for transdisciplinary research to advance knowledge of the efficacy and feasibility of nature-based solutions to sustain the habitability of atolls through a changing climate.

By way of some caveats, the focus of our discussion hereafter is on rural atolls, and on adaptation to sea-level rise. We focus on the direct impacts of sea-level rise because adaptation to avoid its impacts is far more challenging, costly, and uncertain than that to avoid other risks such as, for example, to water supplies or food security, which can be reduced using a suite of standard and proven technologies and practices. We focus on rural atolls, which are by far the majority of all inhabited atolls, because the coasts of most of these atolls are relatively free from substantial human modifications, and nature-based solutions to support adaptation to sea-level rise seem most feasible in such circumstances [14]. Nature-based solutions aim to achieve outcomes using biophysical processes that are free to respond to the changes in conditions affected by climate change, which can be increasingly difficult to implement in islands where there has already been significant coastal armouring that has degraded natural coastal processes [15]. A strategy of further armouring, therefore, seems to be the best option for islands that now have many seawalls and revetments, and the task for adaptation then becomes one of careful design and project management to make these more densely populated islands places where people are able to live dignified and meaningful lives.

It is important to also note here that we do not consider that the efficacy of nature-based solutions should be determined by their potential for carbon sequestration. Atoll societies produce almost no emissions and so hold no legal or moral responsibility for their reduction. Further, despite much talk of ‘blue carbon’ benefits, and the interests of some small island state governments in these, the potential for sequestering carbon in atoll environments is limited by their small size, and the likelihood of significant returns is even further diminished by the low price of carbon on the voluntary market and the transaction costs associated with monitoring and verification in such remote locations [16]. This is not to say carbon sequestration cannot be an incidental benefit of nature-based solutions for atolls, but we do not propose it be a requirement given it can complicate and potentially undermine the efficacy of such solutions for the purposes of adaptation.

## 2. Climate change and the habitability of atolls

Atolls are shallow coral platforms that grow on top of submerged sea-mounts. They are typically formed in a ring surrounding a central lagoon. Atoll islands are composed of carbonate materials deposited from these reefs, and many have sustained human habitation for thousands of years despite having no surface water and porous sandy soils [17]. Inhabited atolls can be found throughout the tropics; the Indian Ocean (56 atolls) and the Pacific Ocean (367 atolls) concentrate 96% of existing world atolls [18]. There are four sovereign countries (Kiribati, the Maldives, the Marshall Islands and Tuvalu) and one dependent territory (Tokelau) comprised entirely of low-lying atolls, and some Pacific Island Countries and Territories (such as French Polynesia) have substantial numbers of inhabited atolls within their territories. Importantly, the geomorphological processes that shape their habitability are not uniform: atolls differ in their size and shape according to mean and extreme waves, winds and local sea-levels, as well as biological processes on reefs and on the land, such that the effect of sea-level rise on atoll landforms will differ [19].

### (a) Risks from sea-level rise

There are cascading uncertainties about climate impacts on atolls that confound adaptation decisions, and which suggest diverse futures are possible. Considering both plausible future emissions scenarios and the response of the Earth system to these, sea-levels in the central Pacific are expected to rise by between 26 and 114 cm by 2100 depending on mitigation scenarios considered in the recent assessment from working group I of the Intergovernmental Panel on Climate Change (IPCC) [20]. This range of uncertainty is far from trivial when considering the potential impacts to atolls, which are rarely more than 2 m above mean-sea-level: adapting to a 26 cm rise in sea-level is a far different proposition than adapting to a 114 cm rise. Yet understanding future sea-levels is only a small part of the knowledge needed to inform adaptation on atolls.

Given that atoll islands are comprised detrital carbonate material that is produced by organisms living on coral reefs, understanding the relationships between reef ecology and mean and extreme sea-levels, sea-surface temperatures and ocean acidity is critical as these are key determinants of island building (as well as the other ecosystem goods and services reefs provide to atoll peoples) [17]. Yet the response of reefs to any of these drivers—let alone their synergistic effects—is not sufficiently understood to enable confident assessments of future states suitable for the purposes of adaptation decision making in any given atoll island. For example, not all reefs can grow with rising sea-levels, but some are more capable than others [21]; rapid changes in sea-surface temperatures cause coral mortality, but not to the same degree in all reefs [22]; and rising ocean acidity will change net calcification on coral reefs, though threshold levels are not well-understood [23]. So, while the future is undoubtedly bad for coral reefs, the ways in which it will be bad, how bad it will be, when impacts will materialize, and what reef ecosystems will look like in the future is uncertain even for the most well-studied coral reefs, let alone for the far less well-studied remote atoll ecosystems.

For the purposes of atoll islands, understanding the relationship between changing reefs and island morphology is also critical. This is the subject of much debate between geologists who typically have a long-term perspective that emphasizes the power of sea-level rise and tends to discount biological processes, and geomorphologists who focus on finer spatial and temporal scales of response [24,25]. The evidence for island responses thus far suggests that few atoll islands are contracting, but many are changing shape [14]. For example, a meta-analysis of published studies of multi-decadal and shoreline changes across 30 atolls comprising 709 islands in the Indian and Pacific oceans found that despite annual changes in sea-level of between 2 and 3.9 mm only 11.4% had contracted, 15.5% increased in area, and 73.1% remained stable, with large islands more likely to be stable or increase in area [26]. Both sides of the debate tend to agree that there will be some degree of self-organization in response to morphodynamical feedbacks such that atoll islands may roll-back towards lagoons, with the question being how many are able to respond in this way, under which conditions, and for how long [27–29]. Some studies indicate larger atolls and wider and higher islands with wider reef flats will experience less erosion, suggesting these may be sites where future investments in critical infrastructure such as airports, hospitals and government centres could be concentrated [25,30,31].

The current debate about island responses is further limited by the absence of locally specific biological information about detrital carbonate sediment budgets, and information about the way changes in currents, waves and winds will alter the magnitude, timing and direction of deposition of calciferous materials on to islands [32,33]. It may be, for example, that high rates of mortality of reef-building species coupled with increasingly frequent high wave events accelerates island building for some time, followed by accelerated erosion as the supply of materials diminishes [34]. The debate is also largely informed by studies of marine processes of deposition, with far less attention given to aeolian processes (but see [35]), and processes driven from oceans across reefs rather than across lagoons (though erosion on lagoon-side shores is also a significant problem). The role of mangroves and seagrasses, which are prevalent in lagoons, is also scarcely considered, though these too play a role in island morphology and habitability [36–38].

### (b) Human drivers of vulnerability

The vulnerability of atoll habitability to climate change is also a function of local anthropogenic drivers that increase both the exposure of socially valued assets and the sensitivity of atoll ecosystems to climate change. For example, in the case of urban atolls population growth can lead to settlement in flood-prone areas, and often poorly sited and constructed causeways, harbours, landing strips, sanitation systems and seawalls. The sensitivity of ecosystems to climate shocks is increased by a range of local human actions—again mostly in urban areas—including the mining of islands and reefs for building materials, the clearing of mangroves, pollution of aquifers from solid and liquid wastes, pollution from land-based sources and ships causing coral morbidity and mortality, damage to reefs from fishing and tourism and disruption of coastal processes from the construction of causeways and seawalls [15,39].

### (c) Adaptation

There is also significant uncertainty about the scope for and limits to societal adaptation on atolls. Adaptation is adjustments in social and environmental systems to avoid or reduce the risks of climate change, and/or to capitalize on new opportunities. Too little is known about what adaptation may entail and can achieve on atolls, because there is far too little investment in research and on tangible initiatives [1,40].

Adaptation can significantly mediate the risks that climate change poses to the habitability of atolls first by reducing social drivers of climate change risks, and then by reducing people's dependence on environmental goods and services that are sensitive to climate change through the use of new technologies and human practices (for example by substituting ground water for improved rainwater harvesting or desalinization, or by substituting ground crops for imported staple foods).

There are examples of highly engineered islands such as Singapore or Hulhumale that have been transformed such that their landforms, water systems, food systems, housing systems and indeed social systems have all been changed in ways that will probably sustain their habitability through a changing climate [41]. There are also a range of typically poorly supplied goods and services that, if improved, could significantly enhance sustainable livelihoods and adaptive capacity in rural islands: these centre around improvements in transport and communications which decrease the costs of and improve access to education and healthcare, and help enable sustainable enterprises such as small-scale tourism operations, value adding to copra and fish production, and pearl farming [42]. It is notable, for example, that by any measure rural poverty in the Maldives is far less than in the Pacific atoll-states, and this can be explained in part by more frequent, faster and cheaper transport services. Thus, there are few hard environmental limits, but many mutable socially derived barriers to adaptation on atolls.

Based on available evidence, there seems to be no limit and few barriers to engineering urban islands such that they remain habitable well beyond this century. The barriers to adaptation are greater for rural atolls, yet it is simply the case that too much still remains unknown about potential impacts and adaptation to make any meaningful estimate of habitability thresholds for such islands (even assuming people's risk thresholds are low and uniform). Considering the scope for adaptation, and that the vast majority of atoll islands have thus far remained stable in size or have increased in size, the habitability of almost all rural atolls should be sustainable well beyond 2050, though this assumes: continued progress in emissions reductions, the low probability extremes of sea-level projected under IPCC low-confidence scenarios do not eventuate, and a far greater commitment to adaptation. As we now explain, the capacity of atoll peoples to adapt should also not be discounted as heavily as it is in much of the literature [43].

### (d) Knowledge of past responses

For as long as they have been settled atoll people have modified their environments to varying degrees, and this has been key to the settlement and continued occupation of these precarious environments, as socio-cultural, demographic and political changes always went hand in hand with ecological ones [44]. The environment of all inhabited atolls is to some degree constructed (more obviously so in urban islands), and this can be seen in landforms that coincide with traditional land and marine tenure patterns, such as gardens where crops are cultivated, pits dug to grow giant swamp taro (*Cyrtosperma chamissonis*), coconut, mangrove and pandanus (*Pandanus tectoris*) stands that stabilize and consolidate shorelines and fishponds and fish traps built for aquaculture [45–47].

Trees in atolls are planted to stabilize the shore, prevent freshwater evaporation in drinking water holes, replenish nutrient-poor soils and, in the case of mangroves, provide nurseries for fish and crustaceans and are a natural buffer against wave action [48]. In Budibudi, a remote atoll in Papua New Guinea, elevated coral rubble mounds have been historically used to plant the few giant taros (*Alocasia macrorrhiza*) that grow in these islands, providing good drainage and preserving crops during flooding events. The people there also build ‘walls’ with coconut fronds to shelter bananas and taro from the sea spray. Similarly, in Kiribati, a type of natural fence known as *te buibui*, made with branches and trunks and held together with sand and coir, is built on the shore as a protective barrier against the wind and seawater [49], with the added benefit of trapping sediment. These responses are those that local people can implement given their means, and their efficacy remains to be seen. They do, however, indicate a propensity for atoll people to work with nature to affect adaptation.

Sea tenure in atolls was characterized by the observance of traditional calendars and cultural practices that regulated the fishing and harvesting of marine produce in the surrounding reefs, making modern marine protected areas redundant [50]. This model also promoted changes to near shore environments in order to manage resources. For example, fishponds and weirs made of coral boulders to trap fish were once common throughout the Pacific [51]. In Budibudi, giant clams (*Tridacnidae* spp.) are collected and brought to the lagoon in proximity of villages, creating farms where juvenile clams are left to mature, providing a readily available source of food particularly rich in vitamin A [52].

Thus, nature-based solutions have been the norm in atolls for a long time, with modern engineering instead being the exception. Nature-based solutions are therefore potentially culturally consistent, especially in rural areas. This and the growing need for adaptation solutions creates a mandate for future research and experimentation and implementation of options that combine traditional practices with modern solutions enabled by advances in environmental science and engineering.

In this context then, there is growing enthusiasm for nature-based solutions to support climate change adaptation. Yet there is very little research focused on this topic, and even less evidence of contemporary practices—at least as reflected in the academic literature. There is certainly nothing like the kind of institutionalization that reinforces the construction of seawalls throughout small island states. There is, however, a growing interest among people within atoll countries that extends from a longer standing interest in marine conservation and ecosystem-based adaptation; for example the Ridge-to-Reef framework [53], and local processes that merge community-based approaches with ecosystem-based approaches, such as Reimaanlok in the Marshall Islands [54]. From such an interest some specific initiatives are emerging, which are being heavily promoted by agencies such as the United Nations Environment Programme and the International Union for Conservation of Nature, and these will help stimulate more widespread recognition and piloting of nature-based solutions across the Indo-Pacific. Nevertheless, it remains the case that there is no community of practice on nature-based solutions in atolls, and nowhere near enough knowledge upon which such a community of practice might emerge.

## 3. Transdisciplinary research for nature-based solutions

The application of nature-based solutions to support people on rural atolls to adapt to sea-level rise requires research that discovers both their effectiveness in providing hazard protection and ecosystem-derived co-benefits, and their feasibility in terms of their social licence, cost and institutionalization. Our goal in this section is to provide a prospectus to help guide research to achieve this goal, a key outcome of which should be the production of rigorous guidelines and design standards for implementing nature-based solutions that can be applied in diverse atoll contexts.

These guidelines and standards should be able to provide broad cost estimates and quantification of the effectiveness of nature-based solutions, because a key barrier to their implementation is a lack of knowledge of their cost-effectiveness in comparison to more traditional hard engineered coastal structures such as seawalls [55]. There is indeed an inversion in the cost-benefit gradient of nature-based solutions as compared to seawalls: the latter have high initial appeal but the infrastructure has increasing ecological and social costs over time [55], whereas the former perhaps have low initial appeal but are supposed to have increasing social and ecological benefits over time [9]. The keys to overcoming this impasse lie in research that demonstrates and properly values the co-benefits that come with nature-based solutions, and which raises awareness of their long-term benefits through evaluation, monitoring, demonstration and dissemination [12,56].

Although recent attempts have been made to commence the development of such guidelines [57], these are only a modest first step towards becoming equivalent to the comprehensive scientific basis and broadly accepted (and often nationally approved) standards that support contemporary engineering approaches such as seawalls, revetments and even sand nourishment. From a practical perspective, the absence of such standards and guidelines creates professional indemnity and public liability risks to implementing agencies and investors, which for now will favour the selection of contemporary engineering options (explicitly as well as indirectly) over nature-based solutions. The presence of standards and guidelines would therefore enable the widespread application of nature-based solutions, which can then become a task for environmental engineering consultants and firms.

The scientific challenges in meeting these objectives are vast, for nature-based interventions require knowledge of the ways in which climatic, oceanographic, geological, morphological and biological processes combine now and into the future to threaten the ability of people to continue to live on atolls—an ability which itself differs according to local cultures, political-economies, technologies and values [58]. As with many contemporary sustainability challenges, successfully tackling these challenges demands a paradigm shift towards transdisciplinary research [59], which organizes research to address societal problems through collaboration between disciplines coupled with collaboration between researchers and stakeholders [60].

In this case the societal problem of concern is the habitability of atolls. The goal of sustaining habitability serves as a meta-objective of research and policy on atolls, providing an answer to the otherwise rarely answered question of ‘adaptation for what purpose?’ [61]. Habitability is also a legitimate objective of adaptation that is shared by atoll peoples and governments: atoll people have a human right to remain on their islands and want the option of remaining on their islands [62–64]. Habitability can also serve as a guiding focus for otherwise diverse strands of research on atolls and on nature-based solutions, which might profitably and usefully consider its implications for atoll habitability.

Transdisciplinary research involves collaboration between disciplines. This is enabled by a shared focus of all disciplines on the societal challenge of atoll habitability, which will be reinforced by the continuous presence of stakeholders (see below). Transdisciplinary research will also be enabled by the necessity of field-based trials of nature-based applications (though modelling will be a necessary first step in many cases). Given that field trials may fail or change the distribution of access to ecosystem goods and services in unforeseen ways, small experiments and experiments in places of lesser social importance seem necessary. Ideal locations include abandoned islands, degraded islands (such as those in the decline phase of the tourism area cycle—[65]) and government-owned islands, though in all cases a degree of co-production will be necessary. These early cases could be established as living laboratories that can serve as demonstration sites and sites for ongoing monitoring [66]. Such trials could focus on three key domains: solutions on reefs, in lagoons and on land.

### (a) Discovering reef-based solutions

It is now reasonably well understood that protection and restoration of coral reefs reduces the risks of extreme events to coastal communities, principally because reef structures formed by corals are naturally efficient at dissipating wave energy [67,68]. Reef restoration can involve outplanting corals or coral fragments on reefs or assisting coral recruitment through various means, such as substratum addition, enhancement or stabilization.

Reef restoration has several benefits, including biodiversity conservation, sustaining reef-dependent livelihoods, helping to attenuate wave energy and contributing to the production of sediments to sustain shorelines. Healthy reefs are also more likely to accrete upwards in response to sea-level rise, and to be more resilient to temperature extremes. It is important to recognize all these benefits when considering the case for and design of nature-based solutions on reefs. For example, if the measure of benefit is only flood mitigation the case for reef restoration seems modest, as suggested in a recent modelling study [69].

Questions remain on how best to undertake coral restoration activities. Most restoration projects to date have been developed from an ecosystem or biodiversity perspective [70]. Consequently, the strategies that have been developed focus on transplanting corals to specific locations [71,72]. However, if a key goal is instead focused on coastal protection, the choice of species and their geographical arrangement as well as their potential growth rate and geometric scale, will become important and affect the anticipated energy dissipation performance. For all this, the capacity of reef restoration projects to sustain large reefs is extremely limited and only likely to have any efficacy under a low-emissions scenario and given early action [73].

The location of reef restoration activities within the reef profile is a key consideration. Targeted applications at smaller scales to sustain specific locations are most likely cheaper than building seawalls and breakwaters, with one study estimating the median cost of artificial breakwaters in the tropics to be US$ 19 791 per m of shoreline enhancement, compared to US$ 1290 for structural coral reef restoration projects [67]. Given that by some estimates the reef crest accounts for 86% of the reduction in ocean wave energy, with the remaining dissipation occurring over the reef flat [67], the focus of nature-based solutions should be on the reef crest, and here recent research on wave energy farms may be relevant. Banks of wave energy generators located along the reef crest can also provide coastal protection benefits by reducing wave energy transmitted to coastlines [74]. With wave-driven coastal flooding along reef coastlines governed by how much wave energy is incident to a reef [75], reductions in offshore wave energy by wave farms could also reduce flooding of atoll islands. In addition, a reduction in wave energy from offshore wave farms could also enhance reef accretion rates [76]. However, much more needs to be known before such a solution can be considered feasible and effective in supporting atoll habitability through a changing climate.

Hybrid approaches that add engineering measures into reef restoration may enhance co-benefits, though there are very few examples of this on coral reefs and atolls. Storlazzi and colleagues modelled the effects of adding a 1 m high solid structure with corals planted on top, to be located either 5 or 25 m from shore, and found that this did not yield significant additional flood risk reduction benefits [69]. Although such structures may not attenuate damage from extreme events, they may yet have benefits for lower-energy sediment movements and for biodiversity in lagoons as well as on reef flats. There is also scope for such structures to contribute to existing as well as new aquaculture projects; for example in the form of cages containing giant clams, or structures that help anchor infrastructure for pearl or fish farms; yet here too there is insufficient knowledge to inform practice.

### (b) Discovering lagoon-based solutions

While coral reefs are critically important for atoll habitability, so too are lagoons. Lagoons are important sources of food in atolls and they can be used for small scale aquaculture (including pearl farming). There are many examples of erosion coming from changes in waves and currents in lagoons that put at risk infrastructure along lagoon shores. In atolls, mangroves are found in the low wave energy and shallow gradients of lagoons, and so may play an important role in mitigating these risks. Where present, and depending on local conditions, mangroves play important roles in facilitating sedimentation that raises the level of the land relative to sea-level, in mitigating wave damage from storms by attenuating wave energy, and as habitats for fishes that support livelihoods [77]. The capacity of mangroves to add to the soil volume by trapping sediments and production of roots allows them to vertically accrete at rates that can keep pace with sea-level rise [78]. There are few measurements of soil surface elevation change in mangroves on atolls, but on Belizean mangrove islands where sediment supply is limited, rates of soil surface elevation gains can reach 5 mm yr^−1^ [79].

Mangroves have other roles in adaptation too; for example, establishing mangroves on sites where there is saline intrusion can help make such areas more economically productive, as well as prevent further erosion—as was the case with the (recently reclaimed) borrow pits in Funafuti (Tuvalu). Aquaculture activities can be better integrated into mangrove forests and their protection. For example, associated mangrove aquaculture [80] or mangrove-based aquaculture [81] seek to restore mangrove green belts along shorelines and waterways, which dampens waves and builds up sediments that in turn protect aquaculture ponds and coastal communities from flooding and inundation. Furthermore, the edible shoots of some mangrove species such as *Bruguiera gymnorhiza* are sometimes used as famine food in atolls in the Maldives and Papua New Guinea.

Removal of mangroves leads to soil subsidence [82], and thus mangrove conservation is widely recognized as being important where mangroves are present. However, where there are benefits in mangrove conservation, the efficacy of new or expanded mangrove stands to support the habitability of atolls is unclear. There are examples where mangroves have been planted in an attempt to protect shorelines but have failed because the conditions were not suitable, for example, in Nanumea in Tuvalu [83]. Further, because mangroves are restricted to low-energy coastal environments, they are unlikely to play a large role in protecting settlements from storm waves in most atolls, although they may provide protection against storm surge [84]. Therefore, while mangroves can in theory provide adaptation and livelihood benefits, the feasibility of these requires systematic research and field trials [85].

There is also growing awareness that marine based aquaculture infrastructure, such as long-line culture of shellfish and seaweeds, can attenuate wave energy, which could be developed in atoll lagoons as an alternative to coastal armouring as a mitigation solution to coastal hazards. The sea pens for other species cultured in the Pacific such as sea cucumbers and milkfish [58], could also be designed and arranged in lagoons to provide similar coastal protection benefits. Again, however, the evidence to support such applications in atolls is scarce, and requires further research.

Seagrass species such as *Zostera marina* are crucial in terms of retaining sediment in atoll lagoons by reducing near-bed flow velocity and wave energy, which minimizes soil erosion, stabilizes coastlines and supports fisheries [86,87]. Of course, in trapping sediments re-established seagrasses may also alter the supply of sediment to islands and so increase erosion. Some studies suggest also they can help enhance coastal protection on barrier reefs [88]. Seagrasses are sensitive to reductions in water quality (high turbidity and nutrient levels) that reduces light level at the ocean floor, and which occurs in lagoons with high human pressures and limited tidal flushing [89]. Additionally, harvesting species from seagrass meadows can also result in compounding damages to seagrass, as evident with the uncontrolled removal of sea cucumbers (*Holothuroidea*) for commercial purposes in Marovo Lagoon (Solomon Islands) [90]. While there are certainly grounds for the conservation of seagrasses, more active interventions, for example restoration through transplanting seagrasses, may also help enhance sediment accretion, reduce wave energy, and assist with food security, though these all require suitable water quality and environmental conditions and careful selection of species type; again, much more research is needed before seagrass restoration could be considered an effective and feasible solution [91].

### (c) Discovering land-based solutions

There is also scope for active land-based interventions to support atoll habitability, notably through the protection, enhancement or restoration of littoral vegetation [92]. There is a long history of the use of vegetation on non-atoll coasts to mitigate the impacts of storms or erosion, though the efficacy of these on atolls is less clear [93,94]. Littoral vegetation can enhance sediment accretion (by trapping sediment either from aeolian deposition or wave deposited sediment) and help mitigate the impact of storms by attenuating wave energy and increasing soil cohesion and so reducing erosion [93,95]. The capacity of any given plant species to perform any of these functions on atolls depends on their growth characteristics. For example, a trait that promotes soil accumulation is the capacity to grow vertically when buried, while stabilization depends on the density of cover and root mass.

Several species common to atoll littoral environments possess traits that promote sediment deposition, stabilize sand or attenuate waves during over wash events (e.g. *Scaevola* sp., *Pandanas* sp., coconuts, *Calophyllym* sp. [92]), however, their effectiveness is still dependent on sediment supply, and empirical evidence for their effectiveness is lacking. There have been relatively few applications of promoting vegetation growth on atoll beaches for erosion mitigation, and the effectiveness of those that have been implemented is not well known [96]. Despite this, there is considerable scope to consider the application of beach rehabilitation techniques that involve vegetation in atoll environments, and this too requires further research.

### (d) Co-production is the key to feasibility

Transdisciplinary research also entails collaboration between researchers and stakeholders, who are regarded as partners rather than consumers of the research [97]. Research on nature-based solutions in atolls must begin with local people and researchers jointly designing the research and then including local and extra-local stakeholders throughout the research process. This is necessary for four principal reasons. First, co-production is an ethical requirement since research on nature-based solutions is not the same as (and is at best a precursor to) implementation; and even when implemented, nature-based solutions may have limited benefits, may not deliver as anticipated, and may indeed have unforeseeable social and ecological impacts about which people need to be fully informed. Failure to co-produce knowledge is therefore likely to lead to false expectations and a legacy of mistrust in nature-based solutions, and in science more broadly. Co-production can also help promote collective solutions that do not exacerbate inequalities in access to environmental goods and services [5,98] since such inequalities do exist even in small remote atoll societies.

A second reason for co-production is because the people who live on atolls have knowledge of environmental processes based on thousands of years of observation and successful management and ignoring this knowledge would be to the detriment of scientific rigour and efficiency. Scientists lacking ‘on the ground’ expertise and unable to spend enough time on atolls can sometimes misread environmental conditions, leading to counterproductive outcomes. Co-production, therefore, helps to ensure a fit between local and ‘western’ knowledge that is necessary to ensure nature-based interventions are well informed.

This then leads to a third reason why co-production helps overcome implementation challenges, at least at the local level [13]. All interventions in the coastal zone face barriers to implementation, including in well-known examples of oyster reefs implemented in liberal-democratic countries with strong policy regimes, alienable property rights and an (modern) ontological distinction between humans and nature [99]. They are perhaps even greater, however, in coastal zones such as those found in atolls which are managed by strong local customary tenure regimes and where the boundaries between land and sea, and people and nature are blurred, and where beliefs, spirits, symbols and totems are important influences [100,101]. In such contexts, co-production helps ensure the solutions being developed are feasible in that they respond to local habitability challenges and values, and fit with local worldviews, decision-making processes, property regimes and beliefs and customs associated with lands and seas [96,102,103].

Finally, coalitions of actors that support nature-based solutions will help catalyse investment in their implementation and institutionalization [6]. Co-production can help ensure the buy-in and alignment of the interests and institutions necessary for the implementation of nature-based solutions on atolls. These interests and institutions include far more than local people, they also include diverse national government agencies responsible for climate change, conservation, development, and planning and who will inevitably be the conduits for funding of nature-based solutions. Thus, research should engage with relevant national agencies throughout the process.

In the case of the Pacific atolls, regional organizations (such as the Pacific Community and the Pacific Regional Environment Programme) are often involved in project funding and implementation and serve as key nodes for information dissemination across multiple countries, so they too should be regularly informed of research on nature-based solutions. Finally, research that helps build the commitment and knowledge of key bilateral and intergovernmental funders of adaptation will help facilitate widespread implementation should nature-based solutions be proved effective and feasible. These donors include the European Union, Japan, and the United States, international non-governmental organizations like the International Union for the Conservation of Nature, and intergovernmental bodies such as the Asian Development Bank, the United Nations' Development and Environment Programmes and The World Bank.

Polycentric governance that includes collaborations across sectors, and scales of organization within them, has been shown to facilitate implementation of nature-based solutions elsewhere, and this is probably also the case in atoll contexts [13]. Nevertheless, researchers should be aware of tensions between local, national and international actors which can impinge on nature-based solutions. For example, some national and international actors may push for projects that generate carbon sequestration credits, which may run counter to local interests, restrict options for adaptation or compromise the adaptation potential of interventions, and incentivize the dispossession of lands and seas from local actors. Good research can therefore help balance power in the institutionalization of nature-based solutions by brokering cross-scale and cross agency partnerships that include local and national governments, civil society, international organizations and the private sector [5].

Thus, to maximize its impact, research on nature-based solutions should be carefully designed with local people, and engage regularly and consistently with national institutions and international actors. This can create demand for nature-based solutions; broker partnerships between international and local actors; maximize the chances of successful field trials; establish engineering, environmental and social standards; and establish processes for evaluation, monitoring and dissemination of best practices. Given the stakes and the rights of local people, such processes are not optional or additional to the research, they are integral and as important as research on the technical aspects of nature-based solutions.

## 4. Conclusion

The survival of atoll societies demands research that can show how nature-based solutions contribute to sustaining the habitability of rural atoll islands through a changing climate in ways that also deliver livelihood and biodiversity co-benefits. These solutions range from reef restoration, artificial structures on reef crests and flats and in lagoons, targeted planting of seagrasses and mangroves, and (re)vegetation of littoral environments. To be effective, such research requires a transdisciplinary approach in which all disciplines collaborate to provide solutions for atoll peoples, and which co-produces knowledge with atoll people and stakeholders.

## Data Availability

This article has no additional data.
